# Device-associated infection and mortality in a Tunisian intensive care unit

**DOI:** 10.1186/2047-2994-4-S1-P208

**Published:** 2015-06-16

**Authors:** N Bouafia, M Mahjoub, I Chouchéne, A Bencheikh, O Ezzi, S Bouchoucha, M Njah

**Affiliations:** 1 Hospital Hygiene, University Hospital Farhat Hached Sousse Tunisia, Jawhara, Tunisia; 2 Medical Intensive Care Unit, University Hospital Farhat Hached Sousse Tunisia, Jawhara, Tunisia

## Introduction

Intensive care unit-acquired infections (ICU-AIs) constitute an important world-wide health problem.

## Objectives

Our aim was to determine the incidence and attributable mortality due to device associated infection (DAI) in ICU patients in Tunisia.

## Methods

We conducted a prospective observational cohort study over a 6 month period in the medical intensive care unit of Farhat Hached University Hospital (Sousse-Tunisia). Patients admitted to the unit were included in the study if they stayed in the ICU for more than 48 hours.

## Results

During the study period 105 patients were surveyed; 16 of them (15.2%) developed 17 episodes of DAI (16.6 DAI/1000 days of hospitalization). The most frequently identified infections were central and peripheral venous catheter-associated infection (respectively 21.4% and 10.2%). At ICU discharge, overall mortality was 40%. Independent risk factors for acquiring infection in ICU were the use of central venous catheter (p=0.014) and length stay, those of mortality in ICU were SAPS II of more than 32.5 points (p=0.003), DAI (p=0.002), central venous catheter (p < 10^-4^) and mechanical ventilation (p=0.04).

## Conclusion

Even if DAI rates in Tunisian ICU were lower than those published in some reports from other North African countries, DAI data, dominated by catheter associated infections show the need for more-effective infection control interventions in our hospital.

## Disclosure of interest

None declared.

